# Prevalence and psychosocial factors associated with serious injuries among in-school adolescents in eight sub-Saharan African countries

**DOI:** 10.1186/s12889-022-13198-6

**Published:** 2022-04-28

**Authors:** Richard Gyan Aboagye, Dickson Okoree Mireku, John Jackson Nsiah, Bright Opoku Ahinkorah, James Boadu Frimpong, John Elvis Hagan, Eric Abodey, Abdul- Aziz Seidu

**Affiliations:** 1 Department of Family and Community Health, School of Public Health, University of Health and Allied Sciences, Hohoe, Ghana; 2 Directorate of Academic Planning and Quality Assurance, University of Cape Coast, Cape Coast, Ghana; 3 Department of Real Estate Management, Takoradi Technical University, Takoradi, Ghana; 4 School of Public Health, Faculty of Health, University of Technology Sydney, Sydney, Australia; 5 Department of Health, Physical Education and Recreation, University of Cape Coast, Cape Coast, Ghana; 6 Neurocognition and Action-Biomechanics-Research Group, Faculty of Psychology and Sport Sciences, Bielefeld University, Bielefeld, Germany; 7 Department of Education and Psychology Studies, University of Cape Coast, Cape Coast, Ghana; 8 Centre for Gender and Advocacy, Takoradi Technical University, Takoradi, Ghana; 9 College of Public Health, Medical and Veterinary Sciences, James Cook University, Townsville, Australia

**Keywords:** Adolescents, Injuries, Sub-Saharan Africa, Public Health, Global School-based Student Health Survey

## Abstract

**Background:**

Injury is one of the major causes of death and illness among children and adolescents worldwide. We sought to investigate the prevalence of serious injury and its associated factors among in-school adolescents in eight countries in sub-Saharan Africa.

**Methods:**

A sample of 14,967 in-school adolescents was drawn from the Global School-based Student Health Surveys conducted from 2012 to 2017 in eight sub-Saharan African countries. Data were collected using self-administered structured questionnaires. The prevalence of serious injuries was calculated using proportions while multivariable binary logistic regression analysis was carried out to determine the factors associated with serious injuries.

**Results:**

Approximately 45% of in-school adolescents had experienced serious injuries during the past 12 months to the survey in the eight sub-Saharan African countries, with variations from 32.3% in Mauritius to 68.2% in Liberia. Adolescents who experienced bullying [aOR = 2.37, CI = 2.10, 2.68], those who engaged in physical fight [aOR = 2.14, CI = [1.87, 2.44], those who experienced an attack [aOR = 1.96, CI = [1.73, 2.22], those who felt anxious [aOR = 1.47, CI = 1.22,1.77], those who attempted suicide [aOR = 1.38, CI = 1.14, 1.65], truants [aOR = 1.33, CI = [1.17,1.51], current tobacco users [aOR = 1.42, CI = [1.01, 2.01] and current marijuana users [aOR = 1.78, CI = 1.08, 2.93] had higher odds of experiencing serious injuries. However, those whose parents or guardians respected their privacy had lower odds of experiencing serious injuries [aOR =0.78, CI = [0.68, 0.88] compared to those whose parents or guardians did not respect their privacy.

**Conclusion:**

A relatively high prevalence of serious injuries among in-school adolescents was identified in the eight sub-Saharan African countries studied. Programs and interventions that target the reduction of injuries in educational institutions should take a keen interest in the factors identified in this study. To deal with injury victims, first aid services should be provided in school settings.

**Supplementary Information:**

The online version contains supplementary material available at 10.1186/s12889-022-13198-6.

## Background

Adolescence, a period of life spanning from 10 to 19 years age, is a unique stage of human development and a crucial time for setting the foundations for good health [1]. According to the World Health Organization (WHO) [1], injuries contribute significantly to the global burden of death and morbidity among children and adolescents [1]. The incidence of injuries among adolescents has already gained much attention because it is classified among the leading factors of disability and death of adolescents in low- and middle-income countries (LMICs) [1, 2]. Three types of injuries are among the top ten causes of disability-adjusted life years (DALYs) for people aged 10 to 24 years, according to the Global Burden of Diseases and Injuries [2]. According to this report, in 2019, traffic injuries (ranked first), self-harm (ranked third), and interpersonal violence (ranked fifth) accounted for 6.6%, 3.7%, and 3.5% of DALYs, respectively.

Past epidemiological studies have shown a 50% reduction in injuries in some industrialized countries over the past 30 years after ‘multisectoral and multipronged approaches to child injury prevention’ were adopted and implemented [3]. Studies have documented the prevalence of injuries among adolescents in several countries. For example, the prevalence of child/adolescent injuries in China was 38.0% [4], 21% in Europe [3], and 24% in Canada [5]. Despite these variations, child injuries remain a problem in several countries [1, 6].

Research report has shown that more than 95% of cases of adolescent injury occur in LMICs and this has negative implications on the physical and psychological health of the victims as well as economic consequences in treating the injury [7]. Ruiz-Casares [8] reported an estimated 53.1/100000 incidence of injuries among adolescents of school-going age in sub-Saharan Africa (SSA). Data from the WHO suggest that a greater proportion of mortality for adolescents aged 10-19 is concentrated in SSA [1]. In the same report, for those aged 10 to 14, mortality ranged from 0.2 to 14.8 deaths per 1000 adolescents aged 10, and for those aged 15 to 19, mortality ranged from 0.8 to 24.9 deaths per 1000 adolescents aged 15, with the majority of these deaths occurring through injuries [1].

To improve adolescent safety in SSA, injury prevention knowledge and practices must be properly integrated into mainstream child and adolescent health initiative programs and policy frameworks. There are multiple sections of the sub-Saharan African region with known high rates of adolescent injury, including Nigeria (74%) [9], Djibouti (61.1%) [10], and Ethiopia (62%) [11], which could potentially adversely affect the rates in other parts of the sub-region.

With the high prevalence of serious injuries in these countries, a comprehensive study that examines the prevalence and correlates of serious injuries among adolescents across several countries in SSA will help to understand the between and within country variations. The present study investigated the prevalence and correlates of serious injuries among in-school adolescents in eight countries in SSA. It is anticipated that the findings would help direct policies aimed at reducing serious injuries among in-school adolescents in SSA.

## Materials and Methods

### Data source and study design

This study utilized data from the Global School-based Student Health Survey (GSHS) of eight sub-Saharan African countries. We included only countries with datasets between 2012 and 2017. The data were obtained from Benin (2016), Ghana (2012), Liberia (2017), Mauritius (2017), Mozambique (2015), Namibia (2013), Seychelles (2015), and Tanzania (2014). The survey employed a cross-sectional design in collecting data from the students. Structured self-administered questionnaires were used to collect data from the students. The GSHS questionnaire collects data on several behavioural risks and protective factors including serious injuries. These factors have the propensity of increasing the students’ risk of morbidities and mortalities. The dataset is freely available at https://extranet.who.int/ncdsmicrodata/index.php/catalog/GSHS

### Sampling method

A two-stage cluster sampling technique was used in sampling the study schools and students for the survey. First, the study schools were selected with probability proportional to the school’s enrolment size. Secondly, classes within the chosen schools were randomly sampled and students aged 10 to 19 in the classrooms of the selected schools were included in the study. The sampling technique used enhanced the random selection of the respondents. Numerical weights were applied to each student record to enable the generalization of results to in-school adolescents. We relied on the “Strengthening the Reporting of Observational Studies in Epidemiology” (STROBE) checklist in  writing the manuscript [12].

### Sample size

A total of 14,967 in-school adolescents aged 10-19 were included in the analysis of this study. Out of this, the sample from each country was Benin (1671), Ghana (2214), Liberia (1167), Mauritius (1995), Mozambique (1033), Namibia (2860), Seychelles (1572), and Tanzania (2455).

### Study variables

#### Outcome variable

The main outcome variable in this study was self-reported serious injury. The question “During the past 12 months, how many times were you seriously injured?” was used to measure the outcome variable. From the GSHS questionnaire, serious injury was defined as an injury that makes the respondent miss at least one full day of usual activities (such as school, sports, or a job) or requires treatment by a doctor or nurse. The response options were 1 = 0 times; 2 = 1 time; 3 = 2 or 3 times; 4 = 4 or 5 times; 5 = 6 or 7 times; 6 = 8 or 9 times; 7 = 10 or 11 times; and 12 or more times. The response options were further categorized into 1 = 0 times [No] and 2 = 1 to 12 or more times [Yes] for this study. The students whose response option was “0 times” showed that they had not sustained any serious injury whilst the remaining response options meant that they had sustained one or more injuries in the 12 months preceding the survey. This categorization has been used in previous studies that utilized the GSHS [13–19]. The detailed question, response option, and coding can be found in the supplementary file attached (S1).

#### Explanatory variables

A total of 22 explanatory variables which had significant associations with injury among in-school adolescents from previous studies [13–19] were considered. These variables were also available in the GSHS datasets. The variables were grouped into sociodemographic characteristics (age, sex, and hunger [a proxy measure of socioeconomic status]), psychosocial environmental factors (current cigarette smoking, current tobacco use, current alcohol use, current marijuana use, anxiety, loneliness, physical fight, physical attack, truancy, suicidal ideation, suicidal plan, suicidal attempt, and bullying), and protective factors (close friends, peer support, parental/guardian supervision, parental/guardian connectedness, parental/guardian bonding, and parental/guardian respect for privacy). The supplementary file attached (S1) has details of the questions, variables, and coding.

### Statistical analyses

**Fig. 1:**
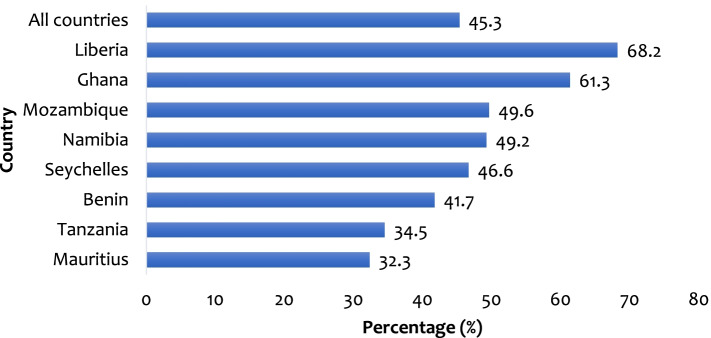
Prevalence of serious injury among the in-school adolescents

Stata software version 16.0 (Stata Corporation, College Station, TX, USA) was used for the data analyses. The prevalence of serious injury among in-school adolescents was presented using proportions (Fig. 1). Pearson’s chi-square test of independence and binary logistic regression models were used to examine the factors associated with serious injury. All the variables with a *p*-value < 0.05 were placed in the regression model. The first model (Model I) consisted of sociodemographic characteristics and serious injury. In the second model (Model II), psychosocial environmental factors were added to the sociodemographic characteristics. The last model (Model III) was controlled for the protective factors and countries. Results of the binary logistic regression were presented as adjusted odds ratios (aOR) with their 95% confidence intervals (CI). Multicollinearity was checked using the variance inflation factor (VIF) and no evidence of high collinearity was found among the studied variables (minimum, maximum, and mean VIF were 1.04, 1.62, and 1.23, respectively). Complex sample analysis (svy) and the inherent sample weight were applied in all analyses to reduce bias from non-response and improve generalizability to all in-school adolescents in SSA.

### Ethical consideration

In the present study, ethics approval was not required since the data were secondary and it is available in the public domain.

## Results

### Descriptive analysis of the prevalence of serious injury among in-school adolescents

The overall prevalence of serious injuries among in-school adolescents in SSA was 45.3% with variations across the countries. This ranged from 32.3% in Mauritius to 68.2% in Liberia (Fig. 1).

### Relationship between serious injury and explanatory variables

Table 1 presents the distribution of serious injury across the explanatory variables. It was observed that all the variables were significantly associated with serious injuries (*p* < 0.05) except the age of the adolescents, close friends, and parental or guardian supervision.

**Table 1:** Bivariate analysis of proportions of serious injury among in-school adolescents in sub-Saharan Africa

Variable	Weighted N	Weighted %	Injured once or multiple times		
			Yes (%)	No (%)	*P*-value
**Age**					0.458
14 years or younger	6082	40.6	45.6	54.4	
15 years or older	8885	59.4	45.1	54.9	
**Sex**					< 0.001
Female	6955	46.5	43.8	56.2	
Male	8012	53.5	46.6	53.4	
**Felt hungry**					< 0.001
No	13,550	90.5	44.2	55.8	
Yes	1417	9.5	55.2	44.8	
**Felt lonely**					< 0.001
No	13,489	90.1	43.8	56.2	
Yes	1478	9.9	59.0	41.0	
**Bullied**					< 0.001
No	9905	66.2	33.8	66.2	
Yes	5062	33.8	67.7	32.3	
**Felt anxious**					< 0.001
No	13,536	90.4	43.3	56.7	
Yes	1431	9.6	63.8	36.2	
**Physical fight**					< 0.001
No	10,597	70.8	36.3	63.7	
Yes	4370	29.2	67.1	32.9	
**Physical attack**					< 0.001
No	8500	56.8	36.0	64.0	
Yes	6467	43.2	57.5	42.5	
**Suicidal ideation**					< 0.001
No	12,918	86.3	43.2	56.8	
Yes	2049	13.7	58.1	41.9	
**Suicidal plan**					< 0.001
No	12,970	86.7	43.2	56.8	
Yes	1997	13.3	58.8	41.2	
**Suicidal attempt**					< 0.001
No	12,916	86.3	42.0	58.0	
Yes	2051	13.7	66.0	34.0	
**Current alcohol use**					< 0.001
No	13,191	88.1	43.7	56.3	
Yes	1776	11.9	57.2	42.8	
**Current cigarette smoking**					< 0.001
No	14,423	96.4	44.3	55.7	
Yes	544	3.6	71.2	28.8	
**Current marijuana use**					< 0.001
No	14,684	98.1	44.6	55.4	
Yes	283	1.9	82.6	17.4	
**Current tobacco use**					< 0.001
No	14,362	96.0	44.0	56.0	
Yes	605	4.0	76.8	23.2	
**Close friends**					0.259
No	1385	9.3	42.5	57.5	
Yes	13,582	90.7	45.6	54.4	
**Truancy**					< 0.001
No	10,825	72.3	40.7	59.3	
Yes	4142	27.7	57.4	42.6	
**Peer support**					< 0.001
No	9818	65.6	47.0	53.0	
Yes	5149	34.4	42.1	57.9	
**Parent or guardian supervision**					0.631
No	7482	50.0	47.1	52.9	
Yes	7485	50.0	43.5	56.5	
**Parent or guardian connectedness**					< 0.001
No	8965	59.9	46.6	53.4	
Yes	6002	40.1	43.3	56.7	
**Parent or guardian bonding**					< 0.001
No	9141	61.1	46.8	53.2	
Yes	5826	38.9	42.9	57.1	
**Parent or guardian respect for privacy**					< 0.001
No	4199	28.1	55.3	44.7	
Yes	10,768	71.9	41.4	58.6	

*P*-values were generated from the chi-square test

### Factors associated with serious injury among in-school adolescents in sub-Saharan Africa

Adolescents who experienced bullying were more likely to report serious injuries compared to their counterparts who were not bullied [aOR = 2.37, CI = 2.10, 2.68]. Higher odds of serious injury were found among adolescents who engaged in physical fight [aOR = 2.14, CI = 1.87, 2.44] and experienced an attack [aOR = 1.96, CI = [1.73, 2.22] as compared to those who did not engage in a physical fight or experienced attacks, respectively. Adolescents who felt anxious [aOR = 1.47, CI = 1.22,1.77] and attempted suicide [aOR = 1.38, CI = 1.14, 1.65] were more likely to experience serious injury compared to those that never felt anxious and never attempted suicide accordingly. The odds of serious injury were higher among truants [aOR = 1.33, CI = 1.17, 1.51], current tobacco users [aOR = 1.42, CI = 1.01, 2.01] and current marijuana users [aOR = 1.78, CI = 1.08, 2.93] compared to non-truant in-school adolescents, non-tobacco users and non-users of marijuana, respectively. However, those whose parents or guardians respected their privacy had lower odds of experiencing serious injuries [aOR = 0.78, CI = 0.68, 0.88] compared to those whose parents or guardians did not respect their privacy (Table 2).

**Table 2:** Factors associated with serious injury among in-school adolescents in sub-Saharan Africa

Variables	Model I aOR [95% CI]	Model II aOR [95% CI]	Model III aOR [95% CI]
**Sex**			
Female	1 [1.00,1.00]	1 [1.00,1.00]	1 [1.00,1.00]
Male	1.11^*^ [1.00,1.23]	1.11 [1.00,1.24]	1.11 [0.99,1.24]
**Felt hungry**			
No	1 [1.00,1.00]	1 [1.00,1.00]	1 [1.00,1.00]
Yes	1.55^***^ [1.31,1.82]	1.15 [0.96,1.38]	1.07 [0.90,1.29]
**Bullied**			
No		1 [1.00,1.00]	1 [1.00,1.00]
Yes		2.78^***^ [2.47,3.13]	2.37^***^ [2.10,2.68]
**Engaged in physical fight**			
No		1 [1.00,1.00]	1 [1.00,1.00]
Yes		2.22^***^ [1.95,2.53]	2.14^***^ [1.87,2.44]
**Physically attacked**			
No		1 [1.00,1.00]	1 [1.00,1.00]
Yes		1.68^***^ [1.50,1.89]	1.96^***^ [1.73,2.22]
**Felt anxious**			
No		1 [1.00,1.00]	1 [1.00,1.00]
Yes		1.55^***^ [1.30,1.86]	1.47^***^ [1.22,1.77]
**Felt lonely**			
No		1 [1.00,1.00]	1 [1.00,1.00]
Yes		1.15 [0.96,1.38]	1.08 [0.90,1.29]
**Suicidal ideation**			
No		1 [1.00,1.00]	1 [1.00,1.00]
Yes		1.02 [0.84,1.24]	1.11 [0.91,1.35]
**Suicidal plan**			
No		1 [1.00,1.00]	1 [1.00,1.00]
Yes		1.13 [0.93,1.37]	1.00 [0.83,1.22]
**Suicidal attempt**			
No		1 [1.00,1.00]	1 [1.00,1.00]
Yes		1.55^***^ [1.30,1.86]	1.37^***^ [1.14,1.65]
**Truant**			
No		1 [1.00,1.00]	1 [1.00,1.00]
Yes		1.39^***^ [1.23,1.58]	1.33^***^ [1.17,1.51]
**Current cigarette smoking**			
No		1 [1.00,1.00]	1 [1.00,1.00]
Yes		1.02 [0.73,1.43]	1.18 [0.82,1.71]
**Current tobacco use**			
No		1 [1.00,1.00]	1 [1.00,1.00]
Yes		1.54^*^ [1.11,2.14]	1.42^*^ [1.01,2.01]
**Current alcohol use**			
No		1 [1.00,1.00]	1 [1.00,1.00]
Yes		1.08 [0.93,1.25]	1.01 [0.85,1.20]
**Current marijuana use**			
No		1 [1.00,1.00]	1 [1.00,1.00]
Yes		1.82^*^ [1.11,2.99]	1.78^*^ [1.08,2.93]
**Peer support**			
No			1 [1.00,1.00]
Yes			1.01 [0.89,1.14]
**Parent or guardian connectedness**			
No			1 [1.00,1.00]
Yes			0.99 [0.87,1.12]
**Parent or guardian bonding**			
No			1 [1.00,1.00]
Yes			0.96 [0.85,1.09]
**Parent or guardian respect for privacy**			
No			1 [1.00,1.00]
Yes			0.78^***^ [0.68,0.88]
**N**	**14,967**	**14,967**	**14,967**
**Pseudo R**^**2**^	**0.003**	**0.136**	**0.158**

Exponentiated coefficients; 95% confidence intervals in brackets; ^*^ *p* < 0.05, ^**^ *p* < 0.01, ^***^ *p* < 0.001; aOR: Adjusted odds ratio;

Model I: Sociodemographic characteristics only

Model II: Adjusted for psychosocial environmental factors

Model III: Adjusted for protective factors and countries

## Discussion

This study examined the occurrence of serious injury among in-school adolescents and its associated factors in eight countries in SSA. The prevalence of serious injuries among in-school adolescents in SSA was 45.3%. The reported prevalence is lower than what has been previously reported in Ghana (57.0%; 66%) [20, 21], Liberia (71.6%) [22], and Nigeria (73.6%) [9], but higher than what was reported in four countries in Asia (36.9%) [15]. A possible reason for this finding could be attributed to the sample size used for the study as well as the scope of the study area. However, there are variations between the country-specific prevalence of serious injuries among in-school adolescents. For instance, while Liberia recorded the highest (68.2%) prevalence, Mauritius on the other hand had the least (32.3%) prevalence. The high-risk violent behaviors among Liberian in-school adolescents which increase their risk of being seriously injured could account for this identified outcome [22].

Corroborating the findings of previous studies [17, 20, 23], the study found that adolescents who experienced bullying were more likely to experience serious injuries than those who were never bullied. From the frustration-aggression perspective, adolescents who are bullied are more likely to retaliate violent behaviors perpetrated against them, causing them to be physically attacked which subsequently increases their probability of sustaining injuries [23–25]. For example, Dollard et al .[26] stated that “the occurrence of aggressive behavior always presupposes the existence of frustration and, contrariwise, that the existence of frustration always leads to some form of aggression” (p. 1). Miller [27] modified the earlier quote by reiterating that “frustration produces instigations to a number of different types of response, one of which is an instigation to some form of aggression” (p. 338).

Adolescents who engaged in a physical fight had a higher tendency of experiencing serious injuries than those who never engaged in a fight. The current finding supports previous studies [20, 23]. Perhaps, the strong association between indulging in aggressive behaviors such as engaging in physical fights and injury sustenance could account for this noted finding [23]. This finding also provides evidence of the strong linkage between physical fights and injury occurrence.

Akin to the findings of other previous studies [18, 20], the study found that adolescents who experienced physical attacks had a higher likelihood of experiencing serious injuries than those who were not physically attacked. It is possible that adolescents who were physically attacked also responded with violent behaviors such as fighting back, making them more likely to sustain serious injuries [18].

The experience of serious injuries was higher among adolescents who felt anxious than those who did not feel anxious. The finding of this study is in line with that of a previous study [21]. Usually, adolescents who are anxious are psychologically distressed and might attempt committing certain self-destructing harm, thus increasing their likelihood of been injured [21, 28]. Such self-destructing harmful behaviors may include suicidal attempt which was also found to increase the likelihood of serious injuries. Similar findings were obtained in previous studies [13, 14, 21]. Smith et al .[29] affirmed that a failed suicide attempt is more likely to result in severe injuries. We also speculate that there is a possibility of bidirectional relationship between anxiety and serious injury in that adolescents that are injured might be more anxious as a result of their injury.

Supporting several previous studies [17, 20–22, 28, 14–16], the current study found that truant adolescents have higher odds of experiencing serious injuries than those who are not truant. Normally, truant adolescents engage in irresponsible behaviors such as street fighting, drug, and alcohol use which increase their likelihood of sustaining injuries [13, 14, 28]. The use of tobacco and marijuana increased the likelihood of serious injuries as corroborated by previous studies [13–15, 17, 21, 22]. An explanation for this finding could be that adolescents who use marijuana or tobacco often engage in violent and aggressive behaviors that predispose them to sustain injuries [22]. It could also be that marijuana has some adverse therapeutic effects that trigger violent and aggressive behaviors among its users, increasing their likelihood of being injured [21].

Parental or guardian respect for privacy was protective against serious injuries among in-school adolescents in SSA. To the best of our knowledge, this is the first study that has found a negative significant association between parental or guardian respect for privacy and serious injuries among in-school adolescents. The observed association could be that the adolescents whose parents respect their privacy might not be anxious that their parents might invade their privacy. As a result, such adolescents are more likely to stay at home and in turn minimize their likelihood of getting injured. Future studies should consider examining why this negative association possibly exists.

### Strengths and limitations

Analyzing data from nationally representative surveys of eight countries in SSA supports the comprehensiveness of the study. Moreover, the secondary data were collected via questionnaires, which allowed many parameters linked with serious injuries to be assessed. Again, the analysis was carried out on a large sample of in-school adolescents and this ensured the accuracy, reliability, and generalizability of the findings. However, there are some limitations to this research that need to be acknowledged. First, the likelihood of social desirability and recall bias cannot be avoided since the assessment of serious injury was based on self-reports. Also, due to the cross-sectional nature of the GSHS, the findings cannot be interpreted using cause and effect. Finally, combining datasets with varying publication years may limit comparisons across countries.  

## Conclusion

A relatively high prevalence of serious injuries among in-school adolescents was identified. The factors associated with serious injuries include bullying, engaging in physical fights, experiencing an attack, anxiety, suicidal attempt, truancy, and substance use. Programs and interventions that target the reduction of injuries in educational institutions should take a keen interest in the factors identified in this study. To deal with injured victims, first aid services should be provided in school settings. Future studies could employ longitudinal designs to assess the association between psychosocial factors and injury.

## Supplementary Information

**Additional file 1.**

## Data Availability

The dataset is freely available at https://extranet.who.int/ncdsmicrodata/index.php/catalog/GSHS.

## Abbreviations

aOR: Adjusted Odds Ratio
CIs: Confidence Intervals
DALYs: Disability Adjusted Life Years
GSHS: Global School-based Student Health Survey
LMICs: Low and Middle-Income Countries
SSA: Sub-Saharan Africa
